# Service user and caregiver involvement in mental health system strengthening in low- and middle-income countries: systematic review

**DOI:** 10.1186/s12913-016-1323-8

**Published:** 2016-03-01

**Authors:** Maya Semrau, Heidi Lempp, Roxanne Keynejad, Sara Evans-Lacko, James Mugisha, Shoba Raja, Jagannath Lamichhane, Atalay Alem, Graham Thornicroft, Charlotte Hanlon

**Affiliations:** King’s College London, Institute of Psychiatry, Psychology & Neuroscience, 16 De Crespigny Park, London, SE5 8AF UK; King’s College London, Faculty of Life Sciences and Medicine, Academic Rheumatology, Clinical Trials Group, Weston Education Centre, 10, Cutcombe Rd., London, SE5 9RJ UK; Butabika National Referral and Teaching Hospital, Kampala, Uganda; Special Advisor, BasicNeeds, 158A Parade, Leamington Spa, Warwickshire, CV32 4AE UK; Nepal Mental Health Foundation, Kathmandu, Nepal; Department of Psychiatry, Addis Ababa University, College of Health Sciences, School of Medicine, Addis Ababa, Ethiopia

**Keywords:** Capacity-building, Service users, Caregivers, Health systems, Mental health, Low- and middle-income countries

## Abstract

**Background:**

The involvement of mental health service users and their caregivers in health system policy and planning, service monitoring and research can contribute to mental health system strengthening, but as yet there have been very few efforts to do so in low- and middle-income countries (LMICs).

**Methods:**

This systematic review examined the evidence and experience of service user and caregiver involvement in mental health system strengthening, as well as models of best practice for evaluation of capacity-building activities that facilitate their greater participation. Both the peer-reviewed and the grey literature were included in the review, which were identified through database searches (MEDLINE, Embase, PsycINFO, Web of Knowledge, Web of Science, Scopus, CINAHL, LILACS, SciELO, Google Scholar and Cochrane), as well as hand-searching of reference lists and the internet, and a snowballing process of contacting experts active in the area. This review included any kind of study design that described or evaluated service user, family or caregiver (though not community) involvement in LMICs (including service users with intellectual disabilities, dementia, or child and adolescent mental health problems) and that were relevant to mental health system strengthening across five categories. Data were extracted and summarised as a narrative review.

**Results:**

Twenty papers matched the inclusion criteria. Overall, the review found that although there were examples of service user and caregiver involvement in mental health system strengthening in numerous countries, there was a lack of high-quality research and a weak evidence base for the work that was being conducted across countries. However, there was some emerging research on the development of policies and strategies, including advocacy work, and to a lesser extent the development of services, service monitoring and evaluation, with most service user involvement having taken place within advocacy and service delivery. Research was scarce within the other health system strengthening areas.

**Conclusions:**

Further research on service user and caregiver involvement in mental health system strengthening in LMICs is recommended, in particular research that includes more rigorous evaluation. A series of specific recommendations are provided based on the review.

**Electronic supplementary material:**

The online version of this article (doi:10.1186/s12913-016-1323-8) contains supplementary material, which is available to authorized users.

## Background

### Rationale

There is wide recognition in principle about the importance of involving service users and their caregivers in health system policy and planning processes, service monitoring and health research. There is also some evidence from high-income countries that their involvement can directly lead to improved mental health system strengthening [1–5]. Participatory policy-making, planning and service monitoring is of particular importance in the field of mental health, where a large majority of people with mental disorders or psychosocial disabilities do not receive any effective treatment or care, and may at times receive treatment against their will [6]. To avoid fragmented and tokenistic inclusion of service users and caregivers in planning, or their marginalisation, and to facilitate meaningful and effective contributions, it is necessary for professionals working within the formal health system to share responsibility with representative organisations. Mental health service user networks have been emerging and are on the increase globally [7, 8]. However, to date there have been very few reports of efforts to involve service users and caregivers in mental health service strengthening in low- and middle-income countries (LMICs). Yet this information is vital in planning capacity-building programmes for service users and caregivers to effectively participate in improving mental health programmes.

This systematic review was undertaken as part of the ‘Emerging mental health systems in LMICs’ (Emerald) programme [9] (see also www.emerald-project.eu), which aims to improve mental health outcomes by generating evidence and capacity to strengthen mental health system performance in six African and Asian LMICs (Ethiopia, India, Nepal, Nigeria, South Africa and Uganda). One of the objectives of the Emerald programme is to empower and equip service users and caregivers to participate more fully and effectively in the mental health system, for example at the level of (i) mental health policy-making and planning processes, (ii) service development and monitoring, and (iii) mental health research. This review served to provide an evidence base to inform the Emerald programme’s capacity-building activities for service users and caregivers.

### Objectives

The objectives for this systematic review were: (i) to systematically synthesise the current evidence and experience base for models of involvement of mental health service users/caregivers in mental health policy-making, mental health service development, quality monitoring and evaluation of services, and mental health research in LMICs; and (ii) to identify models of best practice for evaluation of capacity-building of service users and caregivers to facilitate greater involvement in these mental health system strengthening activities.

## Methods

### Eligibility criteria

The PRISMA checklist is included in Additional file 1. A protocol for the review is available upon request from the authors. Given the scarcity of evidence from LMICs with respect to service user involvement in mental health care more generally, and since initial pilot literature searches carried out by the authors did not identify many relevant papers, a limited number of pertinent resources was anticipated. A broad eligibility framework was therefore adopted, and both peer-reviewed journal papers and grey literature (such as books, unpublished reports, website resources, or training materials) were included in the review.

Whilst the primary focus of the review was capacity-building of service user and caregivers in mental health system strengthening, and the evaluation of capacity-building, a secondary focus was a broader examination of service user and caregiver involvement per se. We therefore included any kind of study design, which reviewed or reported on evaluation or experience of service user (i.e. service users with any kind of mental health problem, including those with intellectual disabilities, dementia, or child and adolescent mental health problems), family or caregiver (though not community) involvement in LMICs, and which were relevant to mental health system strengthening. This entailed direct involvement of service users and caregivers in: (i) development of policies or strategies; (ii) planning or development of services; (iii) training of health workers in mental health care; (iv) service monitoring, evaluation or quality control; or (v) mental health research. Given the dearth of evidence of service user and caregiver involvement in mental health systems, the search was expanded to also include involvement in service delivery and/or support groups, even though their applicability to mental health system strengthening was considered to be limited, unless the support groups contained an advocacy, empowerment or mobilisation component. No studies were excluded based on the type of service, for example whether they were delivered within the formal health system or through alternative avenues.

Although an explicit evaluation of service user/caregiver involvement was required within the original criteria for inclusion, a post hoc decision was made to also include studies that described service user/caregiver involvement in mental health system strengthening according to the categories outlined above, even when there was no clear evaluation strategy. Papers written in English, Spanish, Portuguese, French or German were included. Studies that reported on data solely from high-income countries were excluded.

### Search strategy, study selection and data extraction

To identify relevant peer-reviewed literature, we searched the following databases: MEDLINE [1946 to December 2013], Embase [1974 to December 2013], PsycINFO [1806 to December 2013], Web of Knowledge, Web of Science, Scopus, CINAHL, LILACS, SciELO, Google Scholar and Cochrane [all from the start date of the database to December 2013]. The search strategy is detailed in Table 1.

**Table 1:** Search strategy used in the database search

The following key concepts were used for the search: ‘Service users’ AND ‘health system and services/research’ AND ‘mental health’ AND ‘LMICs’ **Service users** #1 Search: (exp Patient Participation/OR exp Consumer Participation/) OR (patient involvement OR client involvement OR service user involvement OR client participation OR service user participation OR patient participation OR service user engagement OR patient engagement OR service user co-production OR patient co-production).mp. **Health system and services research** #2 Search: (exp Delivery of Health Care/ OR exp Health Policy/ OR exp Health Services/ OR exp Mental Health Services/OR exp Community Mental Health Services/ OR exp Community Health Planning) OR (delivery of health care OR health care delivery OR health system strengthening OR health policy OR health policies OR health system OR health systems OR health services OR mental health system OR mental health systems OR mental health services OR community mental health services).mp. OR Exp Research/ OR research.mp. **Mental health** #3 Search: (exp Mental health/ OR exp Mental Disorders/) OR (“drug abuse” OR “drug addict*” OR “drug depend* *” OR “drug dependence*” OR “drug withdrawal” OR “drug abuse”) OR (“addictive disease*” OR “addictive disorder*”) OR (“alcoholic patient*” OR “alcoholic subject*” OR alcoholism OR “alcohol dependent*” OR “alcohol dependence*” OR “fetal alcohol*” OR “prenatal alcohol*” OR “chronic ethanol*” OR “chronic* alcohol*” OR “alcohol withdrawal” OR “ethanol withdrawal”) OR (“caffeine dependent*” OR “caffeine dependence” OR “caffeine addiction” OR (caffeine AND addict*) OR “caffeine withdrawal”) OR (((cocaine OR heroin OR cannabis OR mdma OR ecstasy OR morphine*) AND (abuse OR depend* OR dependent* OR dependence* OR addict* OR addicts OR addicted OR addiction* OR withdrawal) OR methadone) OR (addiction OR addictive OR “substance abuse” OR “withdrawal syndrome” OR psychoactive*) OR ((schizophrenia OR schizophrenic) OR Schizotyp* OR ((Delusional OR paranoid) AND disorder*) OR hallucination* OR Psychotic OR Schizoaffective OR psychosis) OR (((manic OR bipolar OR mood) AND disorder*) OR (depressive AND (disorder* OR episode*)) OR “depressive symptom*” OR hypomania OR mania* OR ((major OR psychotic OR disorder*) AND depression) OR “suicide attempt*” OR suicidal* OR cyclothymia OR Dysthymia) OR (((anxiety OR panic OR “Obsessive-compulsive” OR adjustment OR conversion OR dissociative OR Somatoform OR Somatization OR neurotic) AND disorder*) OR (“hypochondriasis*” OR “body dysmorphic disorder*” OR “pain disorder*”) OR agoraphobia OR “social phobia*” OR “Post-traumatic stress” OR “stress disorder*”) OR (“Eating disorder*” OR “Anorexia nervosa” OR “Bulimia nervosa” OR “sleep disturbance” OR (sexual AND (disorder* OR dysfunction)) OR ((postnatal OR postpartum) AND depression) OR ((antidepressant* OR laxative* OR analgesic* OR psychotropic* OR vitamin* OR steroids OR hormone*) AND abuse) OR ((insomnia OR sleepiness OR “sleep disturbance”) NOT (apnea OR “side effect*” OR parkinson* OR alzheimer OR neurodegenerat* OR cancer OR obesity OR obese*)) OR (hypersomnia NOT narcolepsy) OR ((sleep OR night) AND terror*) OR nightmare* OR ((disorder* AND (personality OR identity OR impulse* OR impulsive* OR impulsivity)) OR asocial OR antisocial OR psychopathic OR anxious OR narcissi* OR “Pathological gambling” OR pyromania* OR Trichotillomania OR Psychosexual OR (“Munchhausen syndrome”)) OR (“Pervasive developmental disorder*” OR autism OR autistic* OR “Rett* syndrome” OR “Asperger* syndrome”) OR ((Hyperkinetic OR Conduct OR Emotional OR tic) AND disorder*) OR (anxiety AND (separation OR phobic OR social)) OR (hyperactivity AND (disorder* OR syndrome)) OR “Tourette syndrome” OR “ Tourette’s syndrome”) OR ((Mental AND (disorder* OR illness OR health OR health condition OR distress)) OR “psychological distress” OR “psychiatric disorder ”) OR (Nervousness OR “nervous tension” OR Irritability) OR anorexia OR (neurosis OR neuroses OR psychoses) OR ((“mental confusion*”) OR (“mental disability*”) OR (“mental capacity*”) OR ((psychiatric OR mental) AND (comorbidity OR comorbid)) OR psychiatry OR psychology)) **LMICs** #4 Search: (developing OR less developed OR under developed OR underdeveloped OR middle income OR low income OR lower income).mp. AND (countr* OR nation* OR population* or world).mp. OR (transitional OR developing OR less developed OR lesser developed OR under developed OR underdeveloped OR middle income OR low income OR lower income).mp. AND (economy OR economies).mp. OR ((low*).mp. AND (gdp OR gnp OR gross domestic OR gross national).mp.) OR (lmic OR lmics OR lamics OR lamic OR third world OR lami countries OR lami country).mp. OR (transitional country OR transitional countries).mp. OR Exp Developing Countries/ OR (Afghanistan or Albania or Algeria or Angola or Antigua or Barbuda or Argentina or Armenia or Armenian or Aruba or Azerbaijan or Bangladesh or Benin or Byelarus or Byelorussian or Belarus or Belorussian or Belorussia or Belize or Bhutan or Bolivia or Bosnia or Herzegovina or Hercegovina or Botswana or Brazil or Bulgaria or Burkina Faso or Burkina Fasso or Upper Volta or Burundi or Urundi or Cambodia or Khmer Republic or Kampuchea or Cameroon or Cameroons or Cameron or Camerons or Cape Verde or Central African Republic or Chad or Chile or China or Colombia or Comoros or Comoro Islands or Comores or Mayotte or Congo or Zaire or Costa Rica or Cote d Ivoire or Ivory Coast or Croatia or Cuba or Cyprus or Czechoslovakia or Czech Republic or Slovakia or Slovak Republic or Djibouti or French Somaliland or Dominica or Dominican Republic or East Timor or East Timur or Timor Leste or Ecuador or Egypt or El Salvador or Eritrea or Estonia or Ethiopia or Fiji or Gabon or Gabonese Republic or Gambia or Gaza or Georgia Republic or Georgian Republic or Ghana or Gold Coast or Grenada or Guatemala or Guinea or Guam or Guiana or Guyana or Haiti or Honduras or India or Maldives or Indonesia or Iran or Iraq or Jamaica or Jordan or Kazakhstan or Kazakh or Kenya or Kiribati or Korea or Kosovo or Kyrgyzstan or Kirghizia or Kyrgyz or Kirghiz or Kirgizstan or Lao PDR or Laos or Latvia or Lebanon or Lesotho or Basutoland or Liberia or Libya or Lithuania or Macedonia or Madagasca or Malagasy or Malaysia or Malaya or Malay or Sabah or Sarawak or Malawi or Nyasaland or Mali or Marshall Islands or Mauritania or Mauritius or Agalega Islands or Mexico or Micronesia or Middle East or Moldova or Moldovia or Moldovian or Mongolia or Montenegro or Morocco or Ifni or Mozambique or Myanmar or Myanma or Burma or Namibia or Nepal or Netherlands Antilles or New Caledonia or Nicaragua or Niger or Nigeria or Mariana Islands or Oman or Muscat or Pakistan or Palau or Palestine or Panama or Paraguay or Peru or Philippines or Philipines or Phillipines or Phillippines or Romania or Rumania or Roumania or Russia or Russian or Rwanda or Ruanda or Saint Kitts or St Kitts or Nevis or Saint Lucia or St Lucia or Saint Vincent or St Vincent or Grenadines or Samoa or Samoan Islands or Navigator Island or Navigator Islands or Sao Tome or Senegal or Serbia or Montenegro or Seychelles or Sierra Leone or Slovenia or Sri Lanka or Ceylon or Solomon Islands or Somalia or Somaliland or South Africa or Sudan or Suriname or Surinam or Swaziland or Syria or Tajikistan or Tadzhikistan or Tadjikistan or Tadzhik or Tanzania or Thailand or Togo or Togolese or Tonga or Trinidad or Tobago or Tunisia or Turkey or Turkmenistan or Turkmen or Uganda or Ukraine or Uruguay or USSR or Soviet Union or Union of Soviet Socialist Republics or Uzbekistan or Uzbek or Vanuatu or New Hebrides or Venezuela or Vietnam or Viet Nam or West Bank or Yemen or Yugoslavia or Zambia or Zimbabwe or Rhodesia).mp.	

Further relevant literature was identified by: (i) hand-searching reference lists (for example in relevant reviews or journal papers); (ii) a snowballing process of contacting experts active in the area; and (iii) by searching the internet (through use of the Google search engine). Two researchers reached consensus on whether resources that were identified via the grey literature process were eligible for inclusion into the review. Any non-peer-reviewed grey literature found during this search fed into the ‘Discussion’ section of this paper.

For literature that was identified through the database search, each of the titles and abstracts of papers that the search generated were assessed by two independent reviewers. Any paper that was considered to be relevant by either of the two reviewers was included for full-text review. Full-text papers were then screened by two independent reviewers. Where there was disagreement between the independent reviewers of the full-text articles, two senior reviewers made the final decision as to whether the paper fulfilled the inclusion criteria.

A data extraction form (see Tables 2, 3, 4 and 5 for the data obtained from the completed forms) was designed prior to running the review, and the data were extracted by two independent reviewers [one reviewer was used for papers that were written in languages other than English]. The data taken from selected papers was not amenable to statistical synthesis through meta-analysis and was, instead, synthesised into a narrative review, as data were heterogeneous between studies and much of the data were qualitative.

**Table 2:** Overview of quantitative data studies included in the review

Authors	Countries involved	Study design	Participant group and sample size	Area and level of service user involvement	Type of evaluation of involvement (if any)	Outcomes^a^	Summary of findings	Assessment of quality^b^
Aznar et al. (2012) [26]	Argentina and Chile	Development of a scale for the rights of people with intellectual disabilities (ID); cross-sectional comparison between people with ID and controls	37 participants in Delphi group; 51 in pilot study; 705 people with ID in Chile and 524 control University students	People with ID and their families involved in the development of the scale (Delphi group and pilot study)	None	*Other (study: scale development)*: Rights fulfilment score on the devised scale plus 10 individual items on scale; Cronbach’s alpha	The scale may be an appropriate scale to monitor rights, though further development needed. Family relationships, community participation, living arrangements and level of disability affect experience of rights among people with ID. With structured supports, people with ID appear able to exercise their rights to a level comparable to peers without ID.	weak
Malakouti et al. (2009) [22]	Iran	Non-randomised quasi-experimental intervention	12 psychology graduates, plus 9 Consumers’ Family Members (CFM) of people with schizophrenia trained as case managers, of which 6 persons (i.e. 12 in total) from each group were selected as case managers; 129 people with schizophrenia case managed	Training of CFM to be a case management group with 6 family members case managing patients with schizophrenia	CFM group had the potential to be trained as case-managers in mental health, especially if limited resources.	*Service user/caregiver:* Number of hospitalisations of people case-managed by CFMs versus mental health workers plus caregiver burden, knowledge, Quality of Life, general health of caregivers; psychopathology and social skills of schizophrenia patients	Most clinical variables were improved without significant differences between groups. The hospitalization rate was reduced by 67 %. Selection of family of people with severe mental illness should be done with scrutinized criteria considering the refusal rate of 35 % of the subjects in the CFM group (17 % in mental health workers).	weak
McBain et al. (2012) [13]	63 LAMICs and country regions	Data from countries that completed WHO’s Assessment Instrument for Mental Health Systems (WHO-AIMS); multiple regression analyses to investigate role of mental health legislation, human rights implementations, mental health care financing, human resources, and role of advocacy groups on availability and affordability of psychotropic medicines	63 countries/regions, and advocacy groups	Study used ‘yes/no’ questions identifying whether associations of service users or people affected by mental illness were involved in the formulation of mental health legislation	None	*System (country)-level:* Access to psychotropic medicines (availability and affordability) (multiple regression analyses)	Participation of family-based organizations in the development of mental health legislation associated with 17 % greater availability of psychotropic medication	N/A (as between-country comparison rather than individual-level comparison)
Singh et al. (2005) [30]	India	Semi-structured questionnaire on efficiency, punctuality and behaviour of doctors and other staff, waiting time, supply of drugs, and cleanliness of hospital etc.	88 service users and 20 family members from National Drug dependence Treatment Centre Outpatients	Answering of semi-structured questionnaire	Not described	*Other (satisfaction data (service users and caregivers))*: e.g. efficiency, waiting staff, quality of care, general atmosphere, stigmatisation, communication	Over 90 % of patients and their attendants appreciated services provided. 90–94 % were satisfied with the supply of drugs, quality of clinical care and cleanliness of the hospital. Measures for improvement were also suggested.	weak
Tripathy et al. (2010) [23]	India	Cluster-randomised controlled trial	36 clusters in three districts in Jharkhand and Orissa (18 clusters each per intervention and control arm); participants were women who were between 15 to 49 years old, living in the project area, and had given birth during the 3-year study period	Women in intervention clusters participated in groups to support participatory action and learning for women, and to facilitate the development and implementation of strategies to address maternal and newborn health problems	No direct evaluation of involvement, though women’s group intervention included an assessment cycle. Also health committees (with village representatives) and workshops with government health staff included a qualitative assessment by participants at the end of each training session.	*Service user:* Primary outcomes: neonatal mortality rate (NMR); maternal depression scores. Secondary outcomes: stillbirths; maternal and perinatal deaths; uptake of antenatal and delivery services; home care practices during and after delivery; health-care-seeking behaviour.	Women’s groups led by peer facilitators reduced NMR by 32 % during the 3 years overall and by 45 % in years 2 and 3, and moderate maternal depression by 57 % in year 3 (though no significant effect on maternal depression overall), at low cost in largely tribal, rural populations of eastern India.	moderate

^a^Headings in *italics* denote classification of outcomes in terms of ‘system-level’, ‘service user/caregiver’ level, or ‘other’

^b^The ‘Quality assessment tool for quantitative studies’ by the ‘Effective Public Health Practice Project’ (EPHPP) [10, 11] was used for the assessment of quality and risk of bias (see also http://www.ephpp.ca/tools.html). Studies were assessed according to i) likelihood of selection bias; ii) study design; iii) whether confounders were controlled; iv) whether blinding took place; v) validity and reliability of data collection methods; vi) number of withdrawals and drop-outs; vii) intervention integrity; and viii) methods of analyses. A global quality assessment rating of ‘strong’, ‘moderate’ or ‘weak’ was assigned based on the responses within each of those eight categories

**Table 3:** Overview of studies with quantitative and qualitative data that were included in the review

Authors	Countries involved	Study design	Participant group and sample size	Area and level of service user involvement	Type of evaluation of involvement (if any)	Outcomes^a^	Summary of findings	Assessment of quality^b^
Boothby et al. (2011) [14]	Indonesia	Adequacy survey of decentralised mental health services, and outcome study of effect on patients with Axis I mental health disorders	Patients, families, community mental health nurses, sub-district level GPs, volunteer village mental health workers (36 households, number of professionals not specified)	Patients surveyed on their perceived mental health pre- and post-decentralisation of services	None	*Other (perceptions):* Quantitative patient and carer estimations of mental health and wellbeing pre- & post decentralisation; qualitative staff perceptions on functionality of system, adequacy of system	Some progress has been made towards a household-to-hospital continuum of mental health care. Where the system is functioning, it establishes district, sub-district and village levels, which effectively decentralise mental health care services and contribute to community awareness of mental health disorders.	Quantitative data: weak
								Qualitative data:
								Criteria 1, 2, 3, 4, 6, 7, 10, 11, 12: Yes
								Criteria 5, 8, 9: No
Liu et al. (2007) [27]	China	Interviews and surveys of managers of 15 needle exchange programmes, plus interviews with local senior police, peer educators, needles exchange users and patients in compulsory detox	15 managers of needle exchange programmes, plus 15 local senior police, 108 peer educators, 393 needles exchange users, and 86 patients in compulsory detox	Peer educators (majority were active drug users) actively involved in dissemination and needle distribution	Effects of use of peer educators assessed.	*System-level (study) (*quantitative); *Other* (*attitudes) (*qualitative): needle turnover rate, number of clients, attendance, police attitudes, recruitment of peer educators, availability of needles	Needle exchange programmes are improving in terms of needle turnover and attendance. Greater cooperation from police, higher wages for peer educators, and wider awareness of the programmes among drug users are needed to increase coverage. Needle turnover was related to peer educator wages. Peer educators less likely to be arrested. More peer-educators needed.	Quantitative data: weak
								Qualitative data:
								Criteria 1, 2, 3, 4, 10, 11: Yes
								Criteria 5, 6, 7, 8, 9, 12: No
Ndayanabangi et al. (2004) [32]	Uganda	Records review, key informant interviews and focus group discussions to collect data analysed by a cross-section of stakeholders using SWOT system to validate and identify strengths, weaknesses, opportunities and challenges.	Policy makers, health providers and consumers of mental health services (sample size not specified).	Participation in interviews and focus groups	None	*System (country)-level:* Quantitative and qualitative data on country mental health services, e.g. number of mental health professionals, mental health funding, policies and legislation, information systems and research	Mental health service users are rarely informed of their rights, or how to access their records, and rarely make complaints due to ignorance of their rights. Recent development of consumer organisations, e.g. Mental Health Uganda, Ugandan schizophrenia fellowship, Association for parents of children with learning disabilities and Epilepsy support Associations have led to some increased knowledge of consumers in these areas. There is a need to increase advocacy for mental health and develop capacity for professional mental and general health workers supported by appropriate policies, facilities and finances.	Quantitative data: weak (N/A)
								Qualitative data:
								Criteria 1, 2, 10, 11: Yes
								Criteria 3, 4, 5, 6, 7, 8, 9, 12: No

^a^Headings in *italics* denote classification of outcomes in terms of ‘system-level’, ‘service user/caregiver’ level, or ‘other’

^b^For quantitative data, the ‘Quality assessment tool for quantitative studies’ by the ‘Effective Public Health Practice Project’ (EPHPP) [10, 11] was used (see also http://www.ephpp.ca/tools.html) (see Table 2 for further details). For qualitative data, a methodology described by Harden et al. [12] was used (see Table 4 for further details)

**Table 4:** Overview of qualitative data studies included in the review

Authors	Countries involved	Study design	Participant group and sample size	Area and level of service user involvement	Type of evaluation of involvement (if any)	Outcomes^a^	Summary of findings	Assessment of quality^b^
Camatta et al. (2011) [20]	Brazil	Qualitative evaluation of secondary mental health service (in-depth interviews)	13 family members of secondary mental health services	Evaluation of mental health services	Qualitative evaluation of services (rather than of service user involvement) using in-depth interviews. Data were validated in a follow-up workshop with participants	*Other (perceptions/satisfaction):* Interview data were categorised into predefined categories based on both internal and external dimensions of the service. Internal factors included: ambiance, characteristics of the provider team, therapeutic activities and family involvement. External factors included: Public policies (including provision and availability of mental health professionals and treatments), and the relationship between society and mental illness (including better integration of the CAPS service in the community and everyday life).	The article concludes that it is important to give families a voice and to facilitate their collaboration in mental health care and system reform.	Criteria 1, 4, 5, 6, 10, 11: Yes
								Criteria 2, 3, 7, 8, 9, 12: No
Cohen et al. (2012) [25]	Ghana	Qualitative	18 self-help groups (SHGs), 5 NGOs, community mental health nurses, health service administrators	Interviews with these groups/staff	None	*Service user/caregiver:* Clinical, social and economic outcomes, e.g. reasons for joining groups, perceived benefits of membership in groups, social inclusion, social and financial support, biomedical treatments	SHGs have the potential to serve as key components of community mental health programmes in low-resource settings. The strongest evidence concerns how SHGs provide a range of supports, e.g. social, financial, and practical, to service users and caregivers. The groups also appear to foster greater acceptance of service users by their families and by communities at large. Membership in SHGs appears to be associated with more consistent treatment and better outcomes for those who are ill.	Criteria 1, 2, 3, 4, 10, 11, 12: Yes
								Criteria 5, 6, 7, 8, 9: No
Crabtree (2005) [15]	Malaysia (UM)	Ethnographic qualitative methods, in-depth interviews with numerous inpatients using ‘opportunistic sampling’. Staff accounts for insights into the ‘culture’ of hospital setting. Also, critical observation and hospital records over 18 months.	Psychiatric service users, staff (sample size not mentioned)	Interviews with service users	None	*Other (attitudes):* Staff attitudes towards patient ‘compliance’ and resistance to treatment; healing and spirituality	Undisputed power of the medical profession in Malaysia has led to a lack of evolved ‘service-user’ perspective. Few patient rights are recognised, especially non-treatment. Paternalistic and custodial attitude does not acknowledge issues of spirituality/alternative healing practices important to hospitalised patients. Modernisation of services did not lead to parallel development of patient participation/cultural responses.	Criteria 2, 4, 10, 11: Yes
								Criteria 1, 3, 5, 6, 7, 8, 9, 12: No
De La Espriella & Caycedo Bustos (2013) [18]	Colombia	Literature/policy document review and qualitative focus groups and consultation meetings	40 service users, 40 family members and 33 health care professionals	Service user involvement in development of policy/strategy; declaration of mental health patient’s duties and rights	None	*System (study: development of policy):* Qualitative data and document review to develop an institutional policy/declaration of mental health patients’ duties and rights (incl. user participation)	Ten rights/policies were developed/adapted through consultation with service users and families, which ensured comprehensibility, clarity of terms, understanding and sufficient information.	Criteria 1, 2, 4, 5, 10, 11, 12: Yes
								Criteria 3, 6, 7, 8, 9: No
Kleintjes et al. (2013) [28]	Ghana, Kenya, Rwanda, South Africa, Tanzania, Uganda, Zambia	Semi-structured key informant interviews with leaders of mental health self-help organisations, plus documentary review	11 (4 women, 7 men) leaders of 9 self-help organisations for service users and carers	Leaders of self-help organisations interviewed about their experience in the organisations; interview schedule was refined based on feedback from user advocates (and public sector mental health practitioners)	None	*Other (study):* Establishment and sustainability of mental health self-help organisations, e.g. leadership, membership, staffing, advocacy, vision and objectives of organisation	Authors concluded that self-help organisations can provide crucial support to service users’ recovery in resource-poor settings in Africa. Support of other agencies can assist to build organisations’ capacity for sustainable support to members’ recovery.	Criteria 1, 2, 3, 4, 5, 6, 7, 9, 10, 11, 12: Yes
								Criteria 8: No
Nesnanov & Vasilyeva (2013) [31]	Russia	Survey by the Russian Psychiatric Association	Mental health professionals and consumers (sample size not mentioned)	Participation in survey	None	*Other (satisfaction):* Qualitative satisfaction data (on mental health care system)	Majority of professionals and mental health consumers not satisfied with mental health care system in Russia today. Suggestions made to improve services and challenge stigma.	N/A (as congress abstract)
Petersen et al. (2012) [24]	South Africa	Participatory implementation framework for development of mental health services for common mental disorders (CMDs) in a rural sub-district in South Africa as a case study. Qualitative process evaluation by interviewing service providers and users.	Service providers and users (4 focus groups with 15 community mental health workers); 2 interviews with psychosocial group facilitators and 9 participants, 29 community members, 9 representatives from mental health services plus 2 community representatives	Participation in interviews	Involving community members in the development and delivery of psychosocial interventions for women with depression illustrated potential usefulness of community consultation in promoting cultural congruence. Community members well placed to provide local knowledge on interventions to mediate pathways to health and how to manage problems within the constraints of their cultural and material realities. Social support afforded by participation in groups can enhance participants’ individual coping capacities and personal empowerment, supporting previous evidence.	*System, service user/caregiver and other:* Qualitative: 1) benefits and 2) challenges of community participation	In addition to contributing to scaling up mental health services, community participation can potentially promote development of culturally competent mental health services and greater community control of mental health.	Criteria 1, 2, 3, 4, 5, 6, 8, 9, 10, 11, 12: Yes
								Criteria 7: No
Schilder et al. (2004) [29]	Bulgaria (plus exploratory studies in India and Zambia)	Field tests of focus group methodology in India and Zambia with final field test in Bulgaria.	Consumers, family members, NGOs, professionals and government representatives (in Bulgaria: 15 service user, 6 carers, 5 mental health administrators, 11 medical students)	Participation in focus groups	Relatives seemed the most initially eager but dropped out the most.	*Other (study):* Use and appropriateness of focus group methodology	Use of focus groups proved appropriate in helping to clarify issues that could help substantiate data collection and comparison across different cultures and regions. A number of instrument questions were developed further based on the exploratory focus group work.	Criteria 1, 3, 4, 6, 7, 10, 11, 12: Yes
								Criteria 2, 5, 8, 9: No

^a^Headings in *italics* denote classification of outcomes in terms of ‘system-level’, ‘service user/caregiver’ level, or ‘other’

^b^Twelve review criteria were used to assess the quality of qualitative studies. These were based on those suggested in the literature on qualitative research, as described in Harden et al. [12]. The twelve review criteria were as follows: 1. Were the aims and objectives clearly reported? 2. Was there an adequate description of the context in which the research was carried out? 3. Was there an adequate description of the sample and the methods by which the sample was identified and recruited? 4. Was there an adequate description of the methods used to collect data? 5. Was there an adequate description of the methods used to analyse data? 6. Were there attempts to establish the reliability of the data collection tools (for example, by use of interview topic guides)? 7. Were there attempts to establish the validity of the data collection tools (for example, with pilot interviews)? 8. Were there attempts to establish the reliability of the data analysis methods (for example, by use of independent coders)? 9. Were there attempts to establish the validity of data analysis methods (for example, by searching for negative cases)? 10. Did the study use appropriate data collection methods for helping people to express their views? 11. Did the study use appropriate methods for ensuring the data analysis was grounded in the views of people? 12. Did the study actively involve relevant groups in its design and conduct?

**Table 5:** Overview of descriptive non-data-based studies included in the review

Authors	Countries involved	Study design	Participant group and sample size	Area and level of service user involvement	Type of evaluation of involvement (if any)	Type of data collected/outcomes
Agrest (2011) [17]	Argentina (though also discusses historical involvement of service users in other counties (mainly England, Australia, Canada))	Commentary, non-data based paper (opinion/commentary on the history and future of service user groups, especially in relation to Argentina and specifically Buenos Aires)	N/A	There are a range of types of organisations and actors in Buenos Aires related to the service user movement including those related to families/carers, survivors, those attached to human rights, service users only, and also mixed associations with service users, families and psychiatrists. A new movement ‘nothing about us without us’ by and for service users is growing and importantly promotes activities in the community.	N/A	N/A
Ardila (2011) [19]	Argentina	No study design: This paper provides a commentary and develops some ideas related to involving users in service improvement	N/A	N/A	N/A	N/A
Furtado & Campos (2008) [21]	Brazil	Commentary reflection on previous evaluation of mental health service, non-data based paper	N/A	Upon reflection, the participation of service users in the service evaluation was described as ‘gradual’. Researchers were exclusively involved at the start of the project because of funding and time constraints; however, other groups became involved later in the analysis of results, and final workshops and dissemination.	Makes recommendations about what factors to consider in the participatory evaluation of mental health services.	N/A
Hayward & Cutler (2007) [16]	Romania	Describes the progress and achievements of grassroots organisations and people with mental health problems in Romania in developing policies to promote community-based mental health services at the national level.	N/A	Stakeholders from all over Romania had the opportunity to work together, network and create strategic relationships for change by building grassroots coalitions across Romania	This has had some impact on policy-makers and subsequent actions	N/A

### Quality assessment of papers

To assess the quality of papers that were included in the review, for studies containing quantitative data, the ‘Quality assessment tool for quantitative studies’ by the ‘Effective Public Health Practice Project’ (EPHPP) [10, 11] was used (see also http://www.ephpp.ca/tools.html) (see footnote of Table 2 for the eight categories that the methodology includes). A global quality assessment rating of ‘strong’, ‘moderate’ or ‘weak’ was then assigned based on the responses within each of the eight categories. For studies containing qualitative data, twelve review criteria were used, based on those suggested in the literature on qualitative research, as described in Harden et al. [12] (see footnote of Table 4 for the twelve review criteria). Where studies included both quantitative and qualitative data, an assessment was made for both types of data, using the methodologies described above (see Table 3). No quality assessment was made for non-data-based descriptive studies (see Table 5), or for (non-peer-reviewed) grey literature.

### Ethics statement

No ethical review was required as there was no research on human participants.

## Results

### Study selection and characteristics

**Fig. 1:**
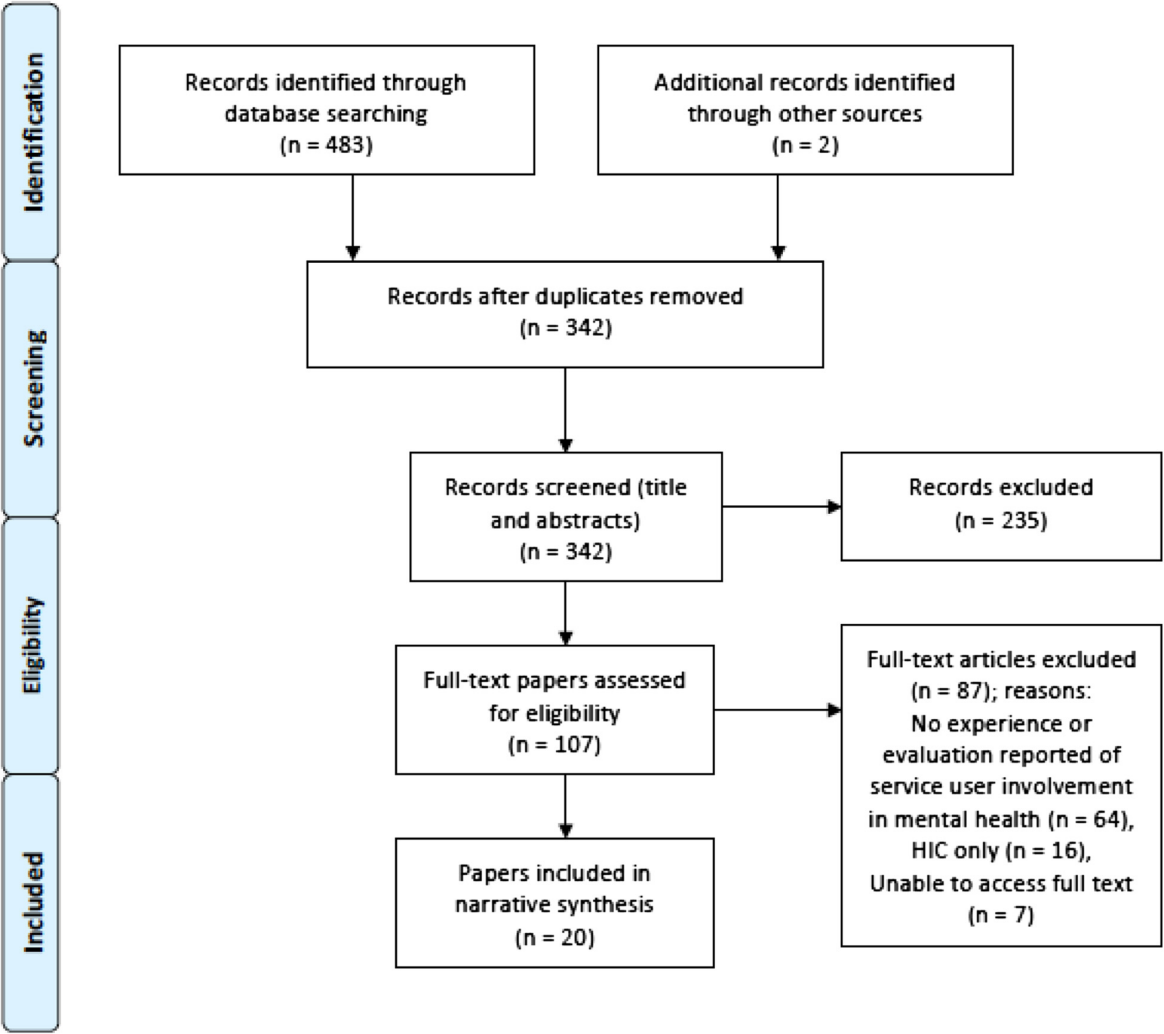
Flow diagram for selection of peer-reviewed articles (format taken from [42])

Figure 1 displays a flow diagram showing the process that was employed in the selection of peer-reviewed articles. In total, 20 papers were included in the narrative review.

The characteristics of the peer-reviewed studies that were eligible for inclusion are shown in Tables 2, 3, 4 and 5. Of the 20 studies, only four included an explicit evaluation of service user/caregiver involvement and thus met our original criteria for inclusion. A further six studies included an evaluation component (though no evaluation of service user or caregiver involvement), six were descriptive data-based studies, and four studies were non-data-based descriptive pieces. Only one (cluster)-randomised controlled trial (RCT) was identified. Across studies, twelve countries were included that were upper-middle income at the time of publication (seven studies from South America, two from Asia, one from Africa, one from Europe, and one from Europe/Asia [Russia]), five lower middle-income countries (three studies from Asia, one from Africa, and one from Europe), and eight countries that were low-income at the time of publication (five from Africa and three from Asia) (according to the World Bank, see http://data.worldbank.org/); a further study included data from 63 different countries or regions [13]. The majority of studies (*n* = 16) included at least one author from a LMIC; four did not [13–16]. Of the 20 papers, eleven were published after 2010, and nine studies were published between 2000 and 2010. Fifteen papers were published in English, three in Spanish [17–19] and two in Portuguese [20, 21].

In accordance with the type of data collected in studies, eight studies included quantitative outcomes and eleven studies included qualitative outcomes/evaluation methods (of which three studies included both types of outcomes/evaluation methods) (see Tables 2, 3, 4 and 5). Of these studies, only four (two quantitative [22, 23] and two qualitative [24, 25]) included outcomes that clearly assessed how service user or caregiver involvement impacted on service users and/or caregivers themselves (for example, in terms of number of hospitalisations, mental health status or coping capacities of service users, caregiver burden). Five studies (two quantitative [26, 27], three qualitative [18, 28, 29]) contained study outcomes that were intended to be improved through service user participation (outcomes included, for instance, needle turnover rates, factors around the sustainability of mental health organisations, development of a scale, establishment of a mental health policy). For six of the studies, the primary outcomes related to satisfaction data or data on perceptions or attitudes (one quantitative [30] and four qualitative studies [15, 20, 27, 31], plus one with both types of data [14]). A further two studies [13, 32] included outcomes at the country level (such as access to medications, number of mental health staff, policies). Four studies did not include any outcomes, as they were non-data-based and descriptive (see Table 5).

In the non-peer-reviewed grey literature, in addition to various websites by service user groups or organisations working with service users or caregivers, five relevant reports were identified, as well as one self-advocacy toolkit, and one newsletter. From these seven sources, information was included from eight low-income countries (Indonesia, Kenya, Lao PDR, Malawi, Nepal, Rwanda, Tanzania, Uganda), three lower middle-income countries (Ghana, India, Sri Lanka), and one upper middle-income country (South Africa), at the time of publication. Two reports were produced after 2010, four between 2005 and 2010, and one before 2000.

### Quality assessment of papers

The majority of included papers (*n* = 8) were qualitative or non-data-based studies (*n* = 4). Five studies presented quantitative data only, with a further three studies including both quantitative and qualitative data. The quality assessment of papers is shown in Tables 2, 3 and 4 for individual studies. Overall, the quality of papers that were included in the review was weak. For studies that included quantitative data, the quality was categorised as weak for all but one paper, for which the quality was categorised to be moderate (the cluster-RCT, which involved peer-facilitated women’s groups, and therefore was one of the papers that was included in the review under the secondary criteria). For papers that included qualitative data, no papers fulfilled all twelve quality assessment criteria, though two papers fulfilled eleven of the criteria. The average number of criteria that were fulfilled was 7.3 (ranging between 4 and 11).

### Findings of peer-reviewed studies

Overall, the studies that were identified in this review provided a weak evidence base for service user and caregiver involvement in mental health system strengthening in LMICs. Many were of low quality (see above) and just four studies included an explicit evaluative element of service user/caregiver involvement. Most of the literature reported about service user and caregiver involvement at the service-level rather than the systems-level, and commonly involved service users as research participants for the evaluation of services rather than their direct participation in the development of policies or services, the training of health workers in mental health care, or within mental health research. There were also few reports that evaluated service user involvement. The level of service user involvement reported in the literature varied considerably across countries. However, there were a few favourable studies that provided some indication as to research areas that could be pursued further, in particular about the development of policies and strategies, including advocacy work, and to a lesser extent the development of services, service monitoring and evaluation.

#### Development of policies or strategies

The main evidence from the peer-reviewed literature in regards to the development of policies or strategies reported on the usefulness and feasibility of consultation processes with service user involvement, and showed that these processes may lead to an improvement in mental health services and/or outcomes. A study that used data from the World Health Organization Assessment Instrument for Mental Health Systems (WHO-AIMS) from 63 countries, for example, showed that participation of family-based organisations in the development of mental health legislation increased availability of psychotropic medication in countries by 17 % [13]. Another paper provided a descriptive account of a consultation process in Colombia with representatives of patients, families, medical students and mental health workers to derive a declaration of mental health patients’ duties and rights [18]. One challenge that has been reported is that people in power may not be willing to give up control, which may result in the exclusion of service users [16]. To address this imbalance in power relations, grassroots public action has been suggested. Hayward & Cutler [16], for instance, described the coalitions between grassroots organisations and people with mental health problems in Romania to develop policies to promote community-based services.

A qualitative study by Ndyanabangi et al. [32] that involved interviews and focus groups with service users, policy makers and health providers in Uganda highlighted the importance, usefulness and feasibility of capacity-building activities for service users and caregivers in advocacy skills. The study established that service users were rarely informed of their rights, so hardly ever made complaints, but that the recent increase in service user organisations had led to an enhanced knowledge base for service users in their rights overall. Similarly, a study in Argentina and Chile found that with structured support, people with intellectual disability (ID) were able to exercise their rights to a level comparable to peers without ID [26]. This may be relevant to policy development in that if service users are not aware or do not have information about their rights, their contribution to policy development is likely to be limited or altogether absent.

#### Planning or development of services

There was very little evidence in the peer-reviewed literature on how best to involve service users and caregivers in the planning or development of services. The best research identified was from a case study on the involvement of community members in the development and delivery of psychosocial interventions for women with depression in South Africa. Through a participatory implementation framework, the service users and service providers were actively involved in the development of the interventions (through in-depth focus groups and consultations), such as a peer facilitated group intervention based on the principles of interpersonal therapy for people with depression [24].

#### Service monitoring and evaluation

The review identified several peer-reviewed papers that involved service users and/or caregivers in the evaluation of mental health services, for example using satisfaction data [14, 20, 31, 33], though few of these included either an evaluation of service user involvement or service user involvement in the monitoring of services themselves. That is, service users or caregivers did not participate in the process of assessing satisfaction levels (monitoring) or devising appropriate responses (service development). However, a useful methodological framework for the evaluation of mental health services was presented in a commentary on the evaluation of mental health services in Brazil, where the participation of service users in the service appraisal was ‘gradual’. That is, researchers were exclusively involved at the start of the project (due to funding and time constraints), but then other stakeholders participated later in the analysis of results, and the final workshops and dissemination [21]. The framework proposed a synthesis of perspectives and recommended five dimensions that should be considered in the participatory evaluation process.

#### Mental health research

The review produced no evidence on how best to involve service users or caregivers within mental health research in LMICs.

#### Training health workers in mental health care

The review produced no evidence or reports on service user or caregiver involvement in the training of health workers in mental health care in LMICs.

#### Service delivery and support groups

The review showed that there is some evidence for the benefits of service user or caregiver involvement in service delivery and/or support groups. This has included involvement of peer educators in needle exchange programmes for alcohol and drug abuse in China [27]; the employment of service users’ family members as case managers for people with schizophrenia in Iran when compared to psychology graduates [22]; service user and carer self-help groups in several African countries [28], such as Ghana [25]; as well as women’s groups led by peer facilitators to reduce moderate maternal depression in India [23].

## Discussion

### Summary of evidence

Overall, this systematic review showed that although there were signs of service user and caregiver involvement in mental health system strengthening in numerous (about 26) countries, there was a lack of high-quality research and a weak evidence base for the work that was being conducted across countries (up to the end of 2013). Most of the literature reported service user and caregiver involvement at the service-level (for example, in regards to the delivery of services such as self-help and support groups) rather than the systems-level (such as at the policy, planning, monitoring or evaluation level), and commonly involved service users as research participants in the evaluation of services (for example, surveying their satisfaction with services) rather than in the direct development of policies or services, the training of health workers in mental health care, or within mental health research. Indeed, no evidence at all was found for the latter two issues, and therefore underlined the substantial gap in the literature in these areas. There were also few reports that evaluated service user involvement. Furthermore, outcomes were often vague in terms of their link to service user involvement; many of the outcomes measured related to perceptions, attitudes or satisfaction data with no clear and direct relation to the impact of service user involvement on mental health system strengthening, particularly in relation to (i) the effects of service user or caregiver involvement on service users or caregivers themselves, or (ii) the impact of their involvement on services.

One reason for these findings may be that most of the service user organisations are still few and fragmented, and that it is difficult to document some of their best practices. However, there were some encouraging studies on the development of policies and strategies, including advocacy work, and to a lesser extent the development of services, service monitoring and evaluation, with some indication to areas that could be pursued in the future.

The review showed overall that service user and caregiver involvement in mental health system strengthening is possible, and there are tentative signs that their direct involvement may lead to improvements in mental health services and outcomes. Generally, research on service user or caregiver involvement in mental health system strengthening seems to be on the rise, as most research has been published in the last ten years. On the other hand, whilst there seem to be good intentions in some countries, these are often not translated into practice. For example, in Argentina, even though there has been an increase in service user involvement over the last twenty years, this has not yet been translated into substantial changes in services or an improvement in service users’ inclusion and quality of life [17]. It was also clear from the literature review that service user and caregiver involvement varied significantly across countries. For instance, whilst in Uganda, quite a large presence of service user advocacy groups is described in the grey literature [8, 34–36], other countries, such as Malaysia, have reported a lack of evolved service user perspectives [15]. Since there are a few countries that have greater awareness in this area than others, it will therefore be important for models of best practice to be shared across countries and regions.

Table 6 provides an overview of recommendations for future studies on service user or caregiver involvement in mental health system strengthening. One important recommendation is for further research to incorporate rigorous evaluative elements of service user and caregiver involvement. This could include high-quality studies such as RCTs, as well as participatory approaches. Case studies that capture the complexity of the impact of service user and caregiver involvement may also yield more revealing insights. Without an evaluation of interventions, the allocation of scarce resources becomes difficult, given the potential for harm (for example, due to stigma), so it is important not to assume that every intervention is beneficial. Future research needs to clarify which interventions are valuable, and also acceptable within different socio-cultural contexts, and which are less so.

**Table 6:** Recommendations for future studies on service user and caregiver involvement in mental health system strengthening

• More high-quality research is needed that directly relates to the systems level (rather than the service-level), specifically to address the gap in evidence on service user and caregiver involvement in the development of policies and strategies, the planning and development of services, the training of health workers in mental health care, and within mental health research. • More systematic evaluation needs to be incorporated into studies of service user and caregiver involvement, including rigorous study designs with low risk of bias, such as RCTs complemented by participatory approaches or case studies. • Outcome evaluations need to be more clearly defined in terms of their relationship to service user or caregiver involvement. Specifically, more studies need to measure the effects of service user or caregiver involvement on either service users or caregivers themselves (e.g. mental health status, well-being, uptake of services, caregiver burden), or the impact of their involvement on services (e.g. availability, accessibility and appropriateness of mental health services, pathways to and through care). • Stakeholder involvement (including service users and caregivers) in study design is recommended that may offer a solution to the slow translation of the findings into meaningful changes in practice at the service or systems level. • Research needs to take into account the local context, culture, traditions and values in the implementation of interventions or capacity-building activities. • Research needs to draw on resources that are available within study countries, e.g. engage and involve policy makers, decision-makers, advocacy or service user groups. Interventions in which there is no or very limited service user involvement may need to focus initially on empowerment or the establishment of new service user groups. • Service users and caregivers need to be fully informed of the reasons for the studies in which they participate and give informed consent to do so. Research could be used as a platform to provide information to service users and caregivers about their rights, and to foster advocacy work. • Models of best practice need to be shared widely and across countries. One way in which to do this may be to empower service user organisations to deliver those services that they are best at, and to then facilitate the documentation of these practices.	

### Grey literature

A search of the grey literature identified several reports of encouraging projects and groups that incorporated participation by service users, their families or caregivers across several countries, particularly in relation to advocacy and empowerment work. A growing number of organisations (in particular non-governmental organisations (NGOs)) across various low- and middle-income countries advocate for service users’ rights and needs, with the recognition that not all service users and families may be interested in roles of activism. These include, for example, Action for Mental Illness (ACMI) in India (see http://mhinnovation.net/organisations/action-mental-illness-india-acmi), who campaign for service user involvement at the social level (for example, running empowerment and advocacy programs for family carers and service users); political level (such as lobbying and negotiating); the legal (state) level; and media level (including civil society and publications). Others include the World Network of Users and Survivors (WNUSP) Working Group (see http://www.wnusp.net/), which was established in 2013 and is a global forum to promote the rights and interests of service users; the Pan-African Network of People with Psychosocial Disabilities (PANUSP) [8] (also see http://www.panusp.org/), which was established in Uganda in 2005 and now extends to nine African countries; and the NGO BasicNeeds, which was founded in 1999 and now has programmes (including empowerment and self-help groups) in at least eight countries across Africa and Asia [37]. A useful toolkit has been developed by BasicNeeds together with the NGO CBM [34], for service users and caregivers who are planning to lead advocacy initiatives. The toolkit was pre-tested by six self-help groups in Uganda (one of the countries that is at the forefront of service user involvement in LMICs), and was peer-reviewed by a wide range of self-advocates and development workers. The toolkit contends that district policy-makers and programme implementers may respond better to advocacy issues that are raised by people affected themselves.

Mental health activists have stressed the importance of taking a holistic approach, in which not only service users, but also caregivers, the surrounding community and decision-makers are engaged, and in which the socio-cultural context is considered. It is also important to take into account the national picture of service user involvement when developing interventions. For example, interventions must consider whether service user groups already exist whose expertise and experiences can be utilised for future involvement, or whether interventions need to focus heavily on empowerment or the establishment of new service user groups. The importance of considering the traditions and values of the socio-cultural context in the implementation of intervention programmes or capacity-building activities (for example, in terms of gender, level of education, or rural–urban cultures) has also been highlighted by advocacy organisations such as ACMI (see above).

In regards to service planning, development and evaluation, a report in the grey literature by TPO (Transcultural Psychosocial Organisation) Uganda [35] described a successful model to scale up mental health services and trauma support in war-affected communities. The process involved service users in the evaluation process (through focus groups and key informant interviews), and patient support groups. The report recommends that services should be monitored and/or evaluated once a year by a wide range of stakeholders, including service users. The use of peer educators for needle exchange programmes for alcohol and drug abuse in Indonesia has also been described in the grey literature [38].

### Limitations

There were several limitations to this review: 1) in regards to the comprehensiveness of studies included, seven studies were excluded because the full-text papers could not be accessed, and one study had to be excluded based on its language of publication (Korean). This may have resulted in a bias in the types of papers that were included in the review (although due to the low number of excluded studies based on these criteria, this bias is unlikely to be substantial). 2) The quality of studies that were included in the review was weak overall, which will have had an impact on the strength of the evidence. This highlights further the need for high-quality research in the area. 3) Systematic reviews are commonly subject to a publication or reporting bias [39–41], whereby only positive outcomes are reported, and unpublished reports are missed. The inclusion of grey literature in the review may have reduced some of this bias, although it is likely that not all eligible resources were identified, given the unsystematic nature of grey literature searches. 4) The review was not listed on an international prospective register of systematic reviews such as PROSPERO (see http://www.crd.york.ac.uk/PROSPERO/), though the original protocol was agreed by all of the co-investigators and is available upon request.

## Conclusions

This systematic review showed that the evidence for how best to involve service users and caregivers in mental health system strengthening in LMICs is not easily accessible in the literature, as well as evidence for the evaluation of user involvement (i.e. how useful or effective user involvement is). Furthermore, despite a few emerging studies, there is still a paucity of high-quality research, especially in regards to service user involvement in the development of policies and strategies, the planning and development of services, the training of health workers in mental health care, and within mental health research. It will therefore be important to develop, test and evaluate models of best practice in the future through rigorous and systematic research. One way in which to do this may be to empower service user organisations to deliver those services that they are best at, which in turn may facilitate the documentation of their best practices.

## Additional file

Additional file 1: PRISMA 2009 Checklist. (DOC 63 kb)

## Abbreviations

ACMI: Action for Mental Illness
Emerald: Emerging mental health systems in low- and middle-income countries
EPHPP: Effective Public Health Practice Project
LMICs: low- and middle-income countries
NGO: non-governmental organisation
PANUSP: Pan-African Network of People with Psychosocial Disabilities
RCT: randomised controlled trial
TPO: Transcultural Psychosocial Organisation
WHO-AIMS: World Health Organization Assessment Instrument for Mental Health Systems
WNUSP: World Network of Users and Survivors
